# COVID-19 vaccination and Atypical hemolytic uremic syndrome

**DOI:** 10.3389/fimmu.2022.1056153

**Published:** 2022-12-01

**Authors:** Romy N. Bouwmeester, Esther M.G. Bormans, Caroline Duineveld, Arjan D. van Zuilen, Anne-Els van de Logt, Jack F.M. Wetzels, Nicole C.A.J. van de Kar

**Affiliations:** ^1^ Radboud University Medical Center, Amalia Children’s Hospital, Radboud Institute for Molecular Life Sciences, Department of Pediatric Nephrology, Nijmegen, Netherlands; ^2^ Radboud University Medical Center, Radboud Institute for Health Sciences, Department of Nephrology, Nijmegen, Netherlands; ^3^ University Medical Center Utrecht, Department of Nephrology and Hypertension, Utrecht, Netherlands

**Keywords:** COVID-19, vaccination, atypical hemolitic uremic syndrome, trigger, complement, SARS-CoV-2, thrombotic microagiopathy, aHUS

## Abstract

**Introduction:**

COVID-19 vaccination has been associated with rare but severe complications characterized by thrombosis and thrombocytopenia.

**Methods and Results:**

Here we present three patients who developed *de novo* or relapse atypical hemolytic uremic syndrome (aHUS) in native kidneys, a median of 3 days (range 2-15) after mRNA-based (Pfizer/BioNTech’s, BNT162b2) or adenoviral (AstraZeneca, ChAdOx1 nCoV-19) COVID-19 vaccination. All three patients presented with evident hematological signs of TMA and AKI, and other aHUS triggering or explanatory events were absent. After eculizumab treatment, kidney function fully recovered in 2/3 patients. In addition, we describe two patients with dubious aHUS relapse after COVID-19 vaccination. To assess the risks of vaccination, we retrospectively evaluated 29 aHUS patients (n=8 with native kidneys) without complement-inhibitory treatment, who received a total of 73 COVID-19 vaccinations. None developed aHUS relapse after vaccination.

**Conclusion:**

In conclusion, aHUS should be included in the differential diagnosis of patients with vaccine-induced thrombocytopenia, especially if co-occuring with mechanical hemolytic anemia (MAHA) and acute kidney injury (AKI). Still, the overall risk is limited and we clearly advise continuation of COVID-19 vaccination in patients with a previous episode of aHUS, yet conditional upon clear patient instruction on how to recognize symptoms of recurrence. At last, we suggest monitoring serum creatinine (sCr), proteinuria, MAHA parameters, and blood pressure days after vaccination.

## Introduction

Atypical hemolytic uremic syndrome (aHUS) is a severe form of thrombotic microangiopathy (TMA) that is characterized by thrombocytopenia, microangiopathic hemolytic anemia (MAHA), and acute kidney injury (AKI) (1–3). Hematologically, TMA can be defined by at least two of the following parameters: platelet count <150 x10^9^/L, lactate dehydrogenase (LDH) serum level above the upper limit of normal, and low or undetectable haptoglobin. Overactivation of the alternative pathway (AP) of the complement system is found to play the main role in the pathophysiology of aHUS and a genetic predisposition of a (likely) pathogenic variant in one or more of the complement (regulatory) proteins, or the presence of anti-complement factor H (CFH) antibodies can be found in up to 70% of aHUS patients (4–6). In aHUS, actual dysregulation of the AP requires a substantial triggering, complement activating event, such as (bacterial and viral) infections, surgery, kidney transplantation, and pregnancy (6, 7). Moreover, in pediatric patients, hepatitis B and diphtheria-pertussis-tetanus-polio (DPTP)) vaccination has been identified as a potential trigger for aHUS (7, 8). To confirm aHUS, a *diagnosis per exclusionem*, it is recommended to perform diagnostics to identify a triggering event, document genetic complement variant(s), and exclude secondary causes of TMA (4, 6, 9). Atypical HUS can be effectively treated with the complement C5-inhibitor eculizumab (which is the only complement-inhibiting therapy available in the Netherlands) (10).

Recently, vaccination campaigns against the coronavirus disease 2019 (COVID-19), also known as severe acute respiratory syndrome coronavirus-2 (SARS-CoV-2), have been implemented worldwide (11). Although rare, COVID-19 vaccination has been associated with severe thrombosis and thrombocytopenia-associated complications (12–14). In addition, a second or third COVID-19 vaccination has been statistically significant associated with (non-aHUS) glomerular disease relapse (15). Sporadically, patients developed complement-mediated HUS after COVID-19 infection and, even more rare, COVID-19 vaccination (14, 16–21). Yet to date it is unknown to what degree vaccination against COVID-19 could trigger aHUS in pediatric and adult patients with a pathogenic complement variant.

Here we describe the association between both Pfizer/BioNTech’s (BNT162b2) mRNA-based and AstraZeneca (ChAdOx1 nCoV-19) adenoviral-based COVID-19 vaccination and aHUS in the Dutch population.

## Method

### Prospective case descriptions

From January 2021 to May 2022 we prospectively identified Dutch pediatric and adult patients who developed onset or relapse aHUS after COVID-19 vaccination with the mRNA-based Pfizer-BioNTech (BNT162b2) or Moderna (mRNA-1273) vaccine, and adenoviral-based AstraZeneca (ChAdOx1 nCoV-19) or Janssen (Ad26.COV2.S) vaccine. Diagnosis of aHUS was made by the treating physician, yet consultation with the aHUS working group was a prerequisite for inclusion in this study. The aHUS working group consists of one pediatric nephrologist and one nephrologist from each University Medical Center in The Netherlands and forms a platform for medical discussions and evaluation of individual cases with (suspected) aHUS. Patients with a confirmed secondary form of TMA and thrombotic thrombocytopenic purpura (TTP, ADAMTS13 activity level <10%) were excluded. In all patients, genomic analysis was performed to screen for variants in complement proteins, including factor H (CFH), factor B (CFB), factor I (CFI), C3, membrane cofactor protein (MCP/CD46), CFH-related proteins 1-5 (CFHR1-5), diacylglycerol kinase-ϵ (DGKE), and thrombomodulin (THBD). Genomic rearrangements in the CFH/CFHR region were assessed using Multiplex Ligation-dependent Probe Amplification (MLPA). In addition, the homozygous presence of at-risk *CFH-H3* and *MCPggaac* haplotypes were evaluated (22, 23). In all patients, detection of autoantibodies against CFH was performed using an in-house enzyme-linked immunosorbent assay (ELISA) (24).

Medical relevant data was obtained from the prospective observational CUREiHUS study (NTR5988), in which all patients were included after giving informed consent. For this study, ethical approval was obtained in the Netherlands from the Medical Research Ethics Committee of Oost-Nederland (NL52817.091.15).

### Retrospective cohort analysis

To estimate the risk of developing aHUS after COVID-19 vaccination, we retrospectively evaluated the clinical course of patients known with aHUS and who received COVID-19 vaccination in the Radboud University Medical Center, the aHUS expertise center of the Netherlands. This cohort was subdivided into patients either treated with or without complement-inhibiting therapy. A potential aHUS relapse was defined as a ≥20% increase in serum creatinine (sCr) after COVID-19 vaccination, and/or ≥2 of the following TMA criteria: thrombocytopenia (platelet count <150 x 10^9^/L), lactate dehydrogenase (LDH) above the upper limit of normal (>250 U/l) and low/undetectable haptoglobin (<0.3 mg/L). This retrospective evaluation required no formal ethical approval nor informed consent, in accordance with Dutch law. Of note, in the majority of patients, laboratory evaluation shortly after vaccination was protocolized due to participation in the RECOVAC study (NL76215.042.2).

### Statistical analysis

Clinical characteristics, including among others medical history and genomic analysis, were descriptively expressed. Laboratory parameters were presented as absolute numbers (for individual values) or median and (min-max) range. Statistical analyses were performed using IBM SPSS Statistics (V.25.0) and figures were drawn using Microsoft Office Excel and Powerpoint (V.2016).

## Results

### Prospective case descriptions

**Figure 1:**
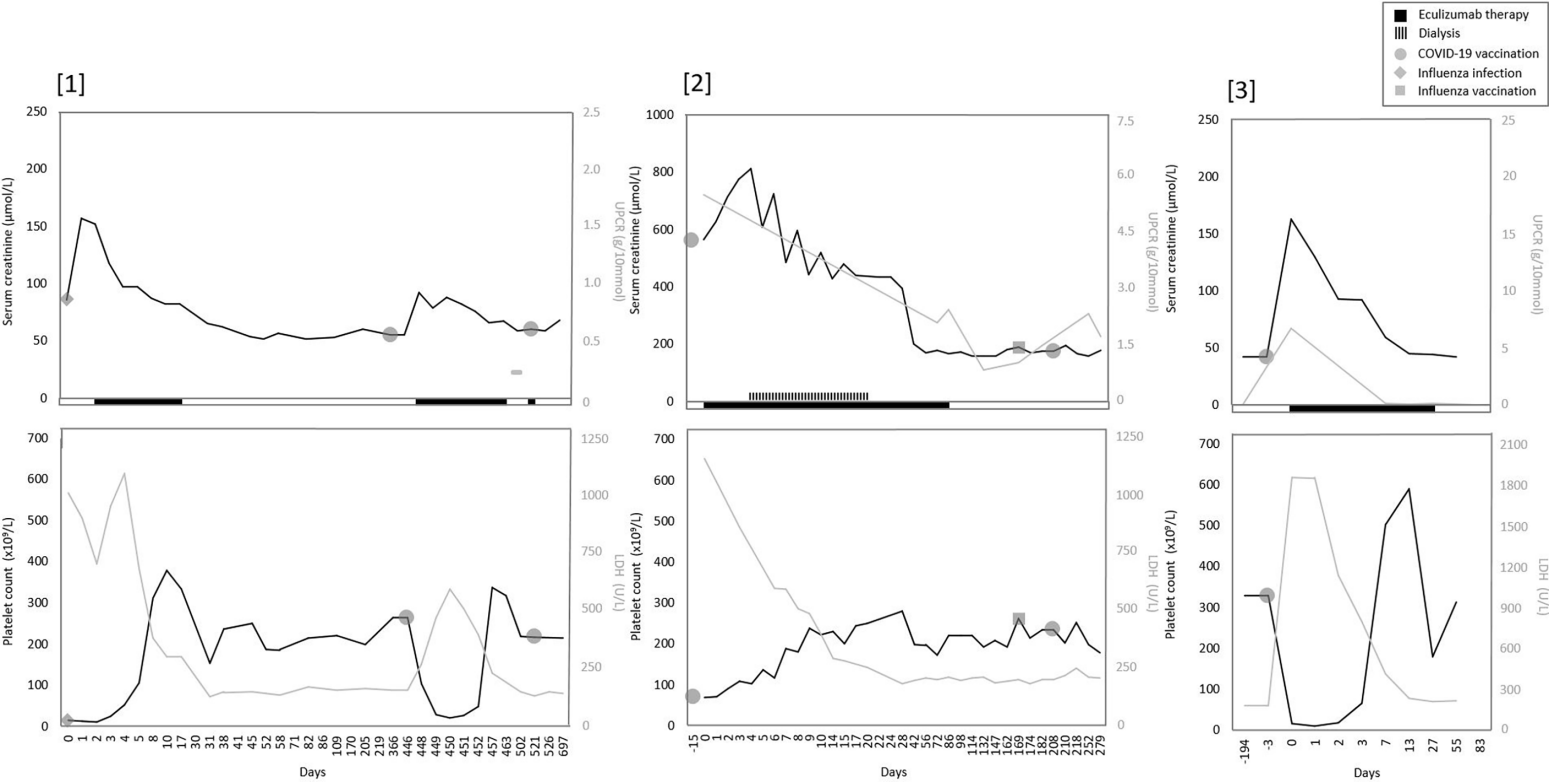
Overview of serum creatinine, proteinuria, and TMA parameters over time in all patients. Overview trend of serum creatinine, proteinuria and TMA parameters LDH and thrombocytes (platelet count). [1] Only one measurement of UPCR was available in patient one. Before the second COVID-19 vaccination, a prophylactic dose of eculizumab was given.

From January 2021 to May 2022, two Caucasian adult patients and one Caucasian pediatric patient, all with the pathogenic C3 variant C.481 C>T (p.(Arg161Trp)) in heterozygosity, developed *de novo* (n=1) or relapse aHUS (n=2) in native kidneys. All three patients presented with evident hematological signs (≥2 parameters) of TMA and AKI (77-560% increase in sCr) (**Figure 1**). In addition, C3 levels were decreased (540 and 650 mg/L, reference range (ref) 700-1500 mg/L) in 2/3 patients (*cases 1* and *2*), but normal (1169 mg/L) in one patient (*case 3*). However, in this patient (*case 3*), levels of C3bBbP (38.0 CAU/ml, ref 0-12), sC5b-9 (0.78 CAU/ml, ref 0-0.5), C3d (15.6 mg/L, ref <8.3) and C3d/C3 ratio (13.3, ref <8.1) were elevated, indicating complement activation. Overall, aHUS was diagnosed a median of 3 days (range 2-15) after mRNA-based (Pfizer/BioNTech’s, BNT162b2) or adenoviral-based (AstraZeneca, ChAdOx1 nCoV-19) COVID-19 vaccination. However, clinical symptoms (other than the most common side-effects after COVID-19 vaccination <48 hours after vaccination) were already reported after a median of 2 (1-3) days. Clinical characteristics and symptoms of all patients are provided in **Table 1**. Of note, in two patients (without complement-inhibitory treatment) no complications occurred after the first COVID-19 vaccination. In addition, the pediatric patient (*case 3*) had been vaccinated according to the Dutch National Immunization Program, yet these vaccinations did not result in aHUS onset.

**Table 1:** Patient Characteristics.

ID	Sex, age^1^ (at onset)	Relevant comorbidities	Native kidneys vs kidney transplant	Genetic complement analysis^2^	Family history	Type of vaccination	Days from vaccination to start of symptoms	Days from vaccination to start of TMA^3^	Clinical signs and symptoms at presentation	Treatment
**Case 1**	Female, 26 (21)	–	Native kidneys	Heterozygous C3 variant C.481 C>T (p.(Arg161Trp))Homozygous haplotype MCP*ggaac*	Yes, aHUS in father and aunt (case 3)	AstraZeneca (ChAdOx1 nCoV-19) Adenoviral vector based vaccine [1^st^]	2	2	Fever, dark urine, ongoing epistaxis, nausea.	Eculizumab (start at TMA day 3) for 2.5 weeks
**Case 2**	Female, 58 (58)	Hypertension, proteinuria	Native kidneys	Heterozygous C3 variant C.481 C>T (p.(Arg161Trp))	Yes, aHUS in sibling and niece (case 1)	AstraZeneca (ChAdOx1 nCoV-19) Adenoviral vector based vaccine [2^nd^]	3	15	Headache, vomiting, dyspnoea on exertion, hypertension, petechiae	Dialysis for 16 days. Eculizumab (start at 1^st^ day of TMA) for 12 weeks
**Case 3**	Male, 12 (10)	–	Native kidneys	Heterozygous C3 variant C.481 C>T (p.(Arg161Trp))	None	Pfizer/BioNTech’sBNT162b2mRNA vaccine [2^nd^]	1	3	Nausea, abdominal pain, jaundice, dark urine, oliguria, petechiae, hypertension	Eculizumab (start at 1^st^ day of TMA) for 4 weeks
**Case 4**	Female, 57 (57)	Crohn’s disease	Native kidneys	Heterozygous C3 variant C.481 C>T (p.(Arg161Trp))Homozygous haplotype MCP*ggaac*	None	Pfizer/BioNTech’s(BNT162b2)mRNA vaccine [1^st^ + 3^rd^]	22nd relapse: 10	72nd relapse: 26	Hypertension and diarrhea	Eculizumab for 12 weeks and (2^nd^ relapse) 15 weeks. Eculizumab was started after respectively 10 and 8 days of TMA.^3^
**Case 5**	Male, 53 (50)	Antiphospholipid syndrome	Native kidneys	Heterozygous C3 variant C.481 C>T (p.(Arg161Trp))Homozygous haplotype MCP*ggaac*	None	Pfizer/BioNTech’sBNT162b2mRNA vaccine [2^nd^]	+/- 40	69 [delay]	Fatigue	Eculizumab (start at TMA day 13) for 12 weeks

1 Age at (first) episode of aHUS after COVID-19 vaccination.

2 Align GVGD, Sorting Intolerant From Tolerant (SIFT) and Polymorphism Phenotyping v2 (PolyPhen) databases/tools were used for prediction of clinical significance. The Genome Aggregation Database (gnomAD) was used to determine the minor allele frequency (MAF). Only variants of unknown significance, (likely) pathogenic variants, and MCPggaac or CFH-H3 risk haplotypes (if present in homozygosity) were reported.

3 Start of TMA was defined as first day of detected laboratory TMA parameters. In case 3, TMA parameters were absent. First day of TMA was defined as start of sCr increase (>15%) after COVID-19 vaccination.

An underlying infection as a trigger for aHUS could be excluded in all patients, as infection parameters were low (CRP <1-7 mg/L, leukocyte count 3.6-10.3x10^9^/L) and viral (among others Influenza A/B and COVID-19) and bacterial (including STEC) serological diagnostics were negative. After eculizumab initiation (within 24 hours after first detection of TMA in 2/3 patients), rapid and complete recovery of TMA parameters was observed in all, which even enabled early (≤5 weeks) eculizumab withdrawal in two patients (*cases 1* and *3*). Kidney function fully recovered to baseline values in two patients but recovery was incomplete in one adult (*case 2*), who required dialysis for a duration of sixteen days in the acute phase. Notably, this latter patient had a medical history of hypertension and was known with proteinuria (UPCR 0.67 g/10mmol). A median of 28 (8-33) weeks after eculizumab discontinuation, kidney function has remained stable in all patients.

In addition to abovementioned patients, two patients with the C.481 C>T C3 variant were treated with eculizumab therapy due to a clinical diagnosis of aHUS relapse after COVID-19 vaccination. Although evident other explanatory factors were absent, in retrospect, in both patients (*cases 4* and *5*) aHUS relapse but also the timely relation with COVID-19 vaccination could be debated (**Table 1**; **Supplementary Figure 1**). One patient (*case 5*) presented with relatively minor changes in TMA parameters and a sCr increase of only 8% (**Supplementary Table 1**; **Supplementary Figure 1**). In addition, TMA parameters were assessed with a delay of 69 days after vaccination and complement activity parameters were not determined. The other patient (*case 4*) was diagnosed with aHUS relapse, but did not present with TMA. This patient was known with chronic mild thrombocytosis due to a relatively small spleen (3.6 centimeters) and/or as a symptom of Crohn’s disease. Although an increase in sCr (18-23%) and UPCR after Pfizer/BioNTech vaccination, TMA parameters all remained within ranges of normal. Complement C3 levels were normal (789 mg/L), but levels of C3bBbP (22.6 CAU/ml), sC5b-9 (0.96 CAU/ml), C3d (8.5 mg/L) and C3d/C3 ratio (10.8) were all mildly elevated, indicating some level of complement activation. In addition, aHUS recurrence could not be excluded due to the presence of unexplained diarrhea (which resembled the clinical picture of her first episode of aHUS), increased blood pressure, and the absence of other explanatory factors for this kidney function decline. In contrast to the abovementioned episodes and despite adequate complement inhibition (eculizumab through level 91µg/mL), in this patient, more evident but self-limiting changes in thrombocytes and LDH levels were observed after the second Pfizer/BioNTech vaccination (**Supplementary Figure 1**, *case 4*).

### Retrospective cohort analysis

Laboratory results after COVID-19 vaccination were available from 29 (including 1 child) and 12 aHUS patients respectively without and with complement-inhibiting therapy, receiving a total of 73 and 31 vaccinations (**Supplementary Table 1**). The majority of patients (21/29 and 8/12) in this retrospective cohort were kidney transplant recipients. In addition, the presence of a complement genetic variant was confirmed in 88% (36/41) of the patients. In all but one patient, TMA was not reported after vaccination. In this one patient, a kidney transplant recipient, ≥2 TMA parameters were found after vaccination(s), however without an increase in sCr and despite adequate complement inhibition (CH50 <10%) with eculizumab. A kidney biopsy was performed, which showed signs compatible with CNI toxicity and no thrombosis. Tacrolimus was discontinued and TMA parameters normalized. Subclinical aHUS recurrence triggered by vaccination was thus deemed unlikely. A ≥20% increase in serum creatinine (sCr) values was found in 6 and 1 patient(s) without and with complement-inhibiting therapy. In all 7 episodes, sCr increase could either be assigned to other explanatory factors (e.g. intercurrent disease) or was temporary, and (re)start of eculizumab was not required. Therefore, clinically relevant aHUS recurrence due to COVID-19 vaccination in these patients was excluded.

## Discussion

We presented two adult patients and one pediatric patient with a pathogenic complement variant, who developed evident new onset or relapse aHUS shortly after either the first, second, or even third COVID-19 vaccination with the mRNA-based Pfizer/BioNTech’s (BNT162b2) or adenoviral-based AstraZeneca (ChAdOx1 nCoV-19) vaccine. To our knowledge, this is the first study to report (COVID) vaccination as a triggering event for aHUS in patients with a documented complement mutation. In all episodes, other aHUS triggering or explanatory events were absent. Atypical HUS onset or recurrence was diagnosed a median of 3 days (range 2-15) after COVID-19 vaccination, but clinical symptoms were reported after a median duration of 2 (1-3) days. In one patient with dubious aHUS relapse, we cannot exclude that aHUS episode could have been diagnosed more profoundly and earlier if TMA parameters were assessed at the time of the start of symptoms. Our data confirms the findings of three adult cases with a pathogenic complement variant, who developed aHUS 1-6 days after BNT162b2, ChAdOx1 nCoV, or mRNA-1273 vaccination (19–21). In addition, two cases of, respectively, a Caucasian and African adult patient who developed TMA onset 5 and 10 days after ChAdOx1 nCoV vaccination have been reported (25, 26). However, aHUS diagnosis in both cases could be debated, as genomic analysis revealed only a benign complement variant in one patient and was not even performed in the other. In addition, in this last patient, clinical symptoms improved by oral prednisolone treatment alone (26).

Last year, the number of reports on thrombocytopenia-associated disorders as a complication of COVID-19 vaccination increased significantly. A vaccine-induced immune thrombotic thrombocytopenia (VITT) was found approximately 5-20 days after AstraZeneca (ChAdOx1 nCoV-19) vaccination (13). VITT is characterized by (cerebral venous sinus) thrombosis, thrombocytopenia, and elevated D-dimer levels. It is caused by the formation of complexes of adenovirus hexon proteins with platelet factor 4 (PF4) on platelet surfaces (27, 28). Subsequently, anti-PF4 autoantibodies are produced and can directly activate platelets (12, 13). Besides VITT, cases of immune thrombocytopenia (ITP) after AstraZeneca (ChAdOx1 nCoV-19) vaccination have been reported, yet antibodies associated with this condition (including anti-PF4 antibodies) have not been identified to date (29, 30). Thrombotic thrombocytopenic purpura (TTP), another form of TMA, has also been associated with both mRNA and adenoviral-based COVID-19 vaccination (31, 32). In 95% of patients, TTP is caused by the presence of anti-ADAMTS13 autoantibodies, and a decreased (<10%) ADAMTS13 activity is found responsible for the low platelet count. Theoretically, TTP after COVID-19 vaccination could be caused by newly-formed autoantibodies. Yet, it is hypothesized that COVID-19 vaccination, especially in early-onset TTP, can be a trigger of occult, undiagnosed TTP rather than inducing TTP. Particularly since ADAMTS-13 antigens and vaccine components (including SARS-CoV-2-spike (S) proteins) bear no resemblance, and the autoimmune process (the formation of autoreactive B-cells, plasma cells, and autoantibodies) and development of the clinical phenotype of TTP requires at least 7-10 days (33).

In aHUS, TMA is the result of overactivation of the alternative complement pathway (AP). COVID-19 infection can potentially trigger aHUS, as COVID-19 initiates an innate immune response (16–18, 34–36). The soluble SARS-CoV-2 spike protein binds to the ACE-2 receptor, expressed on (among others) endothelial cells (37). Subsequently, expression of pro-inflammatory factors (especially TNF-α and IL-1) will induce coagulation and activation of the complement system. In addition, *in vitro*, the SARS-CoV-2 spike protein (subunits 1 and 2) can directly and predominantly initiate the alternative complement pathway (APC) on cell surfaces (33). In their subsequent study, Yu et al. (38) found that dysregulation of the APC plays an important role in disease severity in COVID-19 patients. In example, spike protein 2 directly competes with CFH, and serum from COVID-19 patients could increase membrane attack complex (C5b-9) deposition on cell surface or induce complement-mediated cell death (39). At first, it was not expected that only localized and minor presence of the SARS-CoV-2 spike protein, as expressed after both mRNA and adenoviral-based vaccination, could induce massive acceleration of these mechanisms and thereby cause systemic (pro-thrombotic) conditions in healthy individiduals (13, 33, 39, 40). However, in aHUS patients, the synergistic effect of a local complement-amplifying condition and a pre-existing (pathogenic) complement genetic variant might cause conversion of a ‘normal’ pro-inflammatory state into an unrestrained overactivation of the AP.

Although three cases of aHUS after COVID-19 vaccination appears to be substantial (especially considering a yearly incidence rate in the Netherlands of approximately 10 aHUS episodes), signs of recurrent disease after vaccination were absent in our retrospective cohort of 29 aHUS patients without complement-inhibitory treatment. Of note, this retrospective cohort consisted of patients from (the largest but) a single-center academic hospital in the Netherlands. Unfortunately, the total number of vaccinated aHUS patients in the Netherlands is unknown and data on outpatient clinic visits after vaccination was predominantly available in kidney transplant recipients. All prospective patients (n=5) participated in the CUREiHUS study (in which a total of 49 patients have been included since January 2016), a national Dutch prospective observational study including aHUS patients with a first-time eculizumab treatment since January 2016. Although discussion of all possible aHUS patients in the national aHUS working group for indication to start eculizumab and inclusion in the CUREiHUS study is protocolized in the Netherlands, this is not an absolute prerequisite. Therefore, we cannot exclude missing a patient with aHUS relapse after COVID-19 vaccination in our prospective cohort. It should also be noted that aHUS was only diagnosed after Pfizer/BioNTech’s and AstraZeneca vaccination, yet the majority of patients in our retrospective cohort were vaccinated with Moderna. Due to the small number of patients in both cohorts, an increased risk of aHUS after certain vaccines could not be confirmed nor the definite risk of recurrence be calculated. At last, we could only determine a temporal relationship between COVID-19 vaccination and aHUS, and data on the actual pathophysiological mechanism is not yet available.

In conclusion, we identified COVID-19 vaccination as a potential trigger for aHUS onset or relapse in pediatric and adult patients who are not treated with C5 inhibition. Therefore, aHUS should be included in the differential diagnosis of patients with vaccine-induced thrombocytopenia, especially if co-occurring with MAHA and severe AKI, but in absence of major neurological complications. This underlines the importance of clear patient instruction and routine (laboratory) monitoring after COVID-19 vaccination for kidney function, proteinuria, TMA parameters, and blood pressure days after COVID-19 vaccination. Of note, since the complement system is capricious, uncomplicated previous (COVID-19) vaccinations are no guarantee for future (non-)development of aHUS after vaccination. As the risk of aHUS recurrence after COVID-19 vaccination appears to be acceptable, we advise continuation of COVID-19 vaccination in aHUS patients, as this evidently reduces the risk of severe COVID-19 infection.

## Data availability statement

The original contributions presented in the study are included in the article/**Supplementary Material**. Further inquiries can be directed to the corresponding author.

## Ethics statement

This study involved human participants and was reviewed and approved by the Medical Research Ethics Committee of Oost-Nederland (registration number of the CUREiHUS study: NL52817.091.15) (only applicable for prospective patients). This trial is registered at the Dutch Trial Registry NTR5988. Written informed consent to participate in this study was provided by the participant or participants’ legal guardian/next of kin.

## Author contributions

RB recruited the patients. RB, EB, and CD collected the data. RB and EB wrote the manuscript, under critical supervision and review of AL, AZ, JW, and NK. All authors contributed to the article and approved the submitted version.

## Funding

All prospective patients were included in the CUREiHUS study, which is supported by grants from Zorgverzekeraars Nederland and ZonMw, ‘Goed Gebruik Geneesmiddelen’ (project number 836031008). They did not have any role in data collection, analysis or submission of this manuscript.

## Acknowledgments

We are very grateful to the patients for their willingness to participate in this study. In addition, we thank the research nurses and database managers for their local management and data collection: Yvet Kroeze, Nienke Sonneveld, and Helma Dolmans.

## Conflict of interest

JW is a member of the international advisory board of Alexion and also received a grant from Alexion. NvdK has received a consultancy fee from Roche Pharmaceuticals and Novartis and is a sub-investigator in the APL2-C3G trial, Apellis. AB received a consultancy fee from Novartis, is a member of the DSMB Zoster-047 trial, GSK, and a sub-investigator in the Belatacept study, BMS. VG is sub-investigator in the APL2-C3G trial, Apellis.

The remaining authors declare that the research was conducted in the absence of any commercial or financial relationships that could be constructed as a potential conflict of interest.

## Publisher’s note

All claims expressed in this article are solely those of the authors and do not necessarily represent those of their affiliated organizations, or those of the publisher, the editors and the reviewers. Any product that may be evaluated in this article, or claim that may be made by its manufacturer, is not guaranteed or endorsed by the publisher.
