# Supplementary material for: COVID-19 vaccination and Atypical hemolytic uremic syndrome
Source: Front Immunol. 2022 Dec 1;13:1056153. doi: 10.3389/fimmu.2022.1056153 (PMC9755835; doi:10.3389/fimmu.2022.1056153)
Supplement: Supplementary file 1 [file DataSheet_1.pdf]

Supplementary Table 1. Patient Characteristics and HUS parameters of non-relapsing patients after COVID-19 vaccination

ID	Sex, age[1] at onset, age at vaccination[2]	Genetic complement analysis	Native kidneys vs. Kidney transplant [age at transplant]	Type of vaccine(s)	TMA parameters [day[3]]			AKI parameters [day[3]]		Comments
					Thrombocytes (x10^9/L)	LDH (U/L)	Haptoglobin (g/L)	Creatinine | Last Creatinine[4] (µmol/L)	UPCR | Last UPCR[4] (g/10mmol)	
Vaccinations without complement blockade (CH50 >30%)										
**1**	M, 10y, 13y	C3 C.481C>T p.(Arg161Trp) het.<br>Haplotype MCP*ggaac* homozygous	Native kidneys	Pfizer<br>Pfizer	217 [4]<br>193 [4]	210 [4]<br>224 [4]	0.6 [4]<br>1.0 [4]	58 [4] | 45 [-171][6]<br>54 [4] | 46 [-24]	0.11 [4] | 0.08 [-171]<br>0.10 [4] | 0.11 [-31]	9 days after 1st vaccination creatinine 46 µmol/L
**2**	F, 18y, 36y	C3 C.481C>T (p.(Arg161Trp)) het.	Living donor [21y]	Moderna<br>Moderna	294 [5]<br>277 [28]	256 [4]<br>237 [28]	2.31 [5]<br>1.94 [28]	140 [5] | 171 [0]<br>144 [28] | 141 [0]	0.59 [56] | 0.27 [0]<br>0.59 [28] | 0.27 [-28][5]	
**3**	M, 37y, 62y	MCP c.811_816delGACAGT p.(Asp271_Ser272del) het.<br>Haplotype MCP*ggaac* homozygous	Living donor [50y]	Moderna<br>Moderna<br>Moderna	297 [28]<br>269 [29]<br>298 [28]	195 [64]<br>195 [36]<br>Missing	1.90 [64]<br>1.90 [36]<br>Missing	149 [28] | 122 [-36][6]<br>138 [29] | 149 [0]<br>132 [28] | 117 [-63]	Missing<br>Missing<br>Missing	Day 29 after 1st vaccination creatinine 132 µmol/L
**4**	F, 44y, 46y	C3 c.193A>C p.(Lys65Gln) het.<br>CFI c.1543+5G>T p.(?) het.	Deceased donor [46y]	Pfizer [4th]	304 [17]	252 [17]	Missing	111 [17] | 123 [-11]	0.29 [17] | 0.44 [-11]	Only lab available around 4th vaccination
**5**	F, 23y, 45y	C3 C.481C>T p.(Arg161Trp) het.	Living donor [35y]	Moderna<br>Moderna<br>Pfizer<br>Pfizer<br>Pfizer	207 [48]<br>270 [20]<br>221 [16]<br>278 [28]<br>263 [9]	Missing<br>Missing<br>195 [16]<br>Missing<br>Missing	Missing<br>Missing<br>Missing<br>Missing<br>Missing	147 [48] | 146 [0]<br>147 [20] | 146 [-28][5]<br>122 [16] | 135 [-25]<br>153 [28] | 118 [-52][6]<br>139 [9] | 153 [-84]	Missing<br>Missing<br>Missing<br>Missing<br>Missing	
**6**	F, 28y, 39y	CFH c.3628C>T p.(Arg1210Cys) het.	Living donor [30y]	Moderna<br>Moderna<br>Moderna<br>Moderna	238 [15]<br>222 [29]<br>290 [91]<br>278 [95]	194 [15]<br>209 [29]<br>199 [91]<br>232 [95]	1.67 [15]<br>1.51 [29]<br>1.85 [91]<br>2.00 [95]	86 [15] | 74 [0]<br>89 [29] | 84 [0]<br>81 [91] | 84 [-7]<br>93 [95] | 81 [-3]	0.08 [15] | 0.07 [-34]<br>0.12 [119] | 0.08 [-13]<br>0.14 [189] | 0.11 [-7]<br>0.14 [95] | 0.11 [-101][5]	
**7**	F, 50y, 64y	C3 C.481C>T p.(Arg161Trp) het.	Living donor [55y]	Moderna<br>Moderna<br>Pfizer<br>Pfizer	271 [13]<br>219 [28]<br>289 [94]<br>272 [83]	208 [13]<br>206 [28]<br>206 [94]<br>Missing	1.24 [13]<br>1.33 [28]<br>1.09 [94]<br>0.98 [83]	101 [13] | 154 [0]<br>110 [28] | 112 [0]<br>126 [94] | 109 [-36]<br>113 [83] | 126 [-8]	0.51 [56] | 0.14 [-15]<br>0.51 [28] | 0.14 [-43][5]<br>0.28 [94] | 0.50 [-4]<br>0.34 [83] | 0.28 [-8]	
**8**	M, 38y, 56y	C3 p.Lys65Gln p.(?) het.	Living donor [47y]	Moderna<br>Moderna<br>Pfizer	211 [7]<br>159 [28]<br>178 [7]	213 [7]<br>Missing<br>241 [7]	0.08 [7]<br>Missing<br>0.96 [7]	114 [7] | 134 [0]<br>113 [28] | 117 [0]<br>110 [7] | 110 [-36]	0.08 [7] | 0.12 [0]<br>0.06 [102] | 0.08 [0]<br>0.12 [7] | 0.07 [-36]	

**9**	M, 52y, 66y	C3 C.481C>T p.(Arg161Trp) het.	Living donor [59y]	Moderna<br>Moderna<br>Pfizer<br>Pfizer	196 [14]<br>178 [28]<br>Missing<br>Missing	221 [14]<br>206 [28]<br>310 [35]<br>221 [20]	1.77 [14]<br>1.68 [28]<br>1.50 [35]<br>1.75 [20]	177 [14] | 169 [0]<br>175 [28] | 167 [0]<br>180 [35] | 176 [-21]<br>203 [20] | 180 [-71]	0.28 [14] | 0.36 [-36}<br>0.30 [28] | 0.28 [-14]<br>0.55 [35] | 0.83 [-21]<br>0.85 [20] | 0.55 [-71]	
**10**	F, 18y, 70y	C3 C.481C>T p.(Arg161Trp) het.	Living donor [63y]	Moderna<br>Moderna<br>Pfizer<br>Moderna	194 [14]<br>213 [28]<br>NA<br>216 [3]	252 [14]<br>287 [28]<br>NA<br>306 [3]	1.28 [28]<br>1.52 [28]<br>NA<br>Missing	83 [14] | 82 [0]<br>87 [28] | 71 [0][6]<br>NA<br>92 [3] | 97 [-46]	0.08 [14] | 0.04 [-83]<br>0.07 [79] | 0.08 [-14]<br>NA<br>0.09 [3] | 0.11 [-46]	Values after 3rd vaccination during COVID infection
**11**	F, 16y, 31y	CFH c.1423T>C p.(Tyr475His) het.	Living donor [24y]	Moderna<br>Moderna	199 [28]<br>202 [28]	180 [91]<br>180 [63][5]	1.12 [28]<br>1.05 [112]	106 [28] | 93 [0]<br>96 [28] | 106 [0]	0.10 [91] | 0.12 [0]<br>0.10 [63] | 0.12 [-28][5]	
**12**	F, 63y, 70y	C3 C.481C>T p.(Arg161Trp) het.	Living donor [65y]	Moderna<br>Moderna	166 [14]<br>171 [28]	280 [14]<br>287 [14]	1.32 [14]<br>1.07 [28]	162 [14] | 187 [0]<br>178 [28] | 158 [0]	0.19 [120] | 0.12 [-63]<br>0.19 [120] | 0.12 [-91][5]	
**13**	F, 22y, 40y	No genetic variant<br>Haplotype CFH-H3 homozygous	Living donor [35y]	Moderna<br>Moderna<br>Pfizer	230 [28]<br>243 [20]<br>226 [52]	152 [28]<br>Missing<br>141 [52]	0.98 [28]<br>Missing<br>Missing	76 [28] | 82 [0]<br>80 [20] | 76 [0]<br>76 [52] | 79 [-11]	0.06 [28] | 0.04 [0]<br>0.06 [105] | 0.06 [0]<br>0.09 [52] | 0.08 [-53]	
**14**	M, 36y, 44y	CFH c.158G>A (p.Arg53His) het.	Living donor [41y]	Moderna<br>Moderna	245 [10]<br>234 [24`]	235 [52]<br>235 [24]	1.68 [10]<br>Missing	119 [10] | 109 [-39]<br>123 [24] | 119 [-18]	0.42 [10] | 0.22 [-81]<br>0.33 [80] | 0.42 [-18]	
**15**	F, 47y, 52y	C3 C.481C>T p.(Arg161Trp) het.	Living donor [51y]	Moderna<br>Moderna	192 [7]<br>207 [23]	231 [7]<br>203 [65]	Missing<br>Missing	125 [7] | 126 [-21]<br>120 [23] | 125 [-19]	0.14 [189] | 0.17 [-21]<br>0.14 [163] | 0.17 [-47][5]	
**16**	F, 48y, 54y	No genetic variant	Living donor [54y]	Moderna<br>Moderna<br>Moderna	151 [6]<br>157 [7]<br>173 [9]	255 [0]<br>175 [7]<br>174 [9]	2.07 [6]<br>1.47 [7]<br>2.29 [9]	95 [6] | 92 [-7]<br>92 [7] | 95 [-7]<br>122 [7] | 113 [-31]	0.12 [0] | 0.15 [-7]<br>0.13 [7] | 0.12 [-21]<br>0.19 [7] | 0.18 [-26]	
**17**	F, 42y, 45y	No genetic variant	Deceased donor [42y]	Moderna<br>Moderna<br>Moderna	286 [7]<br>245 [28]<br>189 [25]	203 [21]<br>219 [45]<br>180 [25]	0.65 [21]<br>0.88 [45]<br>0.59 [25]	NA<br>NA<br>NA	NA<br>NA<br>NA	Patient on dialysis
**18**	F, 12y, 24y	No genetic variant	Living donor [15y]	Moderna<br>Moderna<br>Pfizer	198 [5]<br>218 [39]<br>183 [16]	196 [5]<br>177 [39]<br>Missing	Missing<br>Missing<br>Missing	195 [5] | 176 [5]<br>192 [39] | 195 [-17]<br>250 [16] | 215 [-54]	1.55 [5] | 1.75 [-37]<br>0.84 [39] | 0.89 [-17]<br>2.46 [16] | 1.11 [-54]	Kidney function deterioration independent of vaccination
**19**	F, 36y, 59y	CFH c.1520-1G>A p.(Glu566Ala) het.	Deceased donor [58y]	Pfizer [3rd]	250 [12]	282 [12]	2.28	92 [12] | 92 [-16]	0.39 [5] | 0.23 [-16]	First 2 vaccinations during eculizumab treatment (below)
**20**	F, 39y, 55y	C3 C.481C>T p.(Arg161Trp) het.	Deceased donor [49y]	Moderna<br>Moderna<br>Pfizer	156 [28]<br>139 [7]<br>146 [63]	159 [28]<br>155 [7]<br>204 [63]	1.28 [28]<br>Missing<br>0.96	138 [28] | 115 [0][6]<br>108 [7] | 138 [0]<br>84 [63] | 80 [-20]	0.12 [35] | 0.16 [-29]<br>0.12 [7] | 0.16 [-57][5]<br>0.56 [63] | 0.20 [-34]	Day 35 after 1st vaccination sCr 108 µmol/L
**21**	M, 39y, 63y	C3 C.481C>T p.(Arg161Trp) het.	Deceased donor [58y]	Moderna<br>Moderna	173 [80]<br>173 [52][5]	208 [80]<br>208 [52][5]	0.56 [80]<br>0.56 [52][5]	99 [80] | 89 [-25]<br>99 [52] | 89 [-53][5]	0.05 [80] | 0.04 [-68]<br>0.05 [52] | 0.04 [-96][5]	

				Pfizer	167 [42]	213 [42]	0.63 [42]	112 [42] | 106 [-42]	0.05 [42] | 0.06 [-42]	
**22**	F, 29y, 33y	CFH c.1548T>A p.(Asn516Lys) het.<br>Haplotype CFH-H3 homozygous	Living donor [32y]	Pfizer [4th]	246 [36]	181 [36]	0.85 [36]	94 [36] | 89 [-2]	Missing	First 3 vaccinations during eculizumab treatment (below)
**23**	M, 33y, 38y	C3 C.481C>T p.(Arg161Trp) het.	Native kidneys	Moderna [1st]	189 [4]	197 [4]	1.48 [4]	93 [4] | 92 [-79]	0.06 [4] | 0.08 [-79]	2nd vaccination after 1 eculizumab administration
**24**	F, 17y, 36y	CFH c.1198C>A p.(Gln400Lys) het.<br>MCP c.811_816del (p.Asp271_Ser272del) homo.	Native kidneys	Moderna [1st]	302 [9]	168 [9]	1.47 [9]	164 [9] | 183 [-18]	1.48 [9] | 0.59 [-25]	2nd and 3rd vaccinations under eculizumab
**25**	F, 41y, 50y	CFI c.355G>A p.(Gly119Arg) het.<br>Haplotype CFH-H3 homozygous	Native kidneys	Pfizer [3rd]	229 [14]	159 [14]	2.30 [14]	225 [14] | 222 [-154]	Missing	First 2 vaccinations during eculizumab treatment (below)
**26**	F, 58y, 65y	CFI c.355GA p.(Gly119Arg) het.<br>CFI c.7723GA p.Ala258Thr het.	Native kidneys	AstraZen.<br>AstraZen.<br>Moderna<br>Moderna	224 [42]<br>248 [7]<br>187 [1]<br>198 [6]	223 [42]<br>254 [7]<br>230 [1]<br>233 [6]	1.20 [42]<br>1.10 [7]<br>1.30 [1]<br>1.40 [6]	212 [42] | 233 [-70]<br>199 [7] | 212 [-35]<br>243 [1] | 214 [1]<br>225 [6] | 229 [-49]	Missing<br>Missing<br>Missing<br>Missing	
**27**	F, 64y, 65y	C3 c.3760C>T p.(Arg1254Cys) het.	Native kidneys	AstraZen.<br>AstraZen.<br>Pfizer	263 [14]<br>236 [14]<br>237 [52]	199 [14]<br>216 [14]<br>Missing	0.60 [14]<br>0.80 [14]<br>0.60 [52]	90 [14] | 101 [-56]<br>82 [14] | 85 [-14]<br>112 [52] | 83 [-18][6]	Missing<br>Missing<br>Missing	
**28**	M, 45y, 45y	CFI c.1650_1651del p.(Cys550Trpfs*17) het.	Native kidneys	Moderna<br>Moderna	325 [7]<br>231 [28]	274 [7]<br>Missing	Missing<br>Missing	NA<br>NA	NA<br>NA	Patient on dialysis. 2rd vaccination under eculizumab
**29**	F, 55y, 55y	Missing	Native kidneys	Pfizer [3rd]	252 [11]	238 [11]	1.0 [11]	571 [11] | 516 [-6]	Missing	Only lab available around 3rd vaccination
Vaccinations under complement blockade (CH50 <30%)										
**ID**		**Genetic complement analysis**			**TMA parameters [day[3]]**			**AKI parameters [day[3]]**		**Comments**

	Sex, age[1] at onset, age at vaccination[2]		Native kidneys vs. Kidney transplant [age of transplant]	Type of vaccine(s)	Thrombocytes (x10^9/L)	LDH (U/L)	Haptoglobin (g/L)	Creatinine | Last Creatinine[4] (µmol/L)	UPCR | Last UPCR[4] (g/10mmol)	
**19**	F, 36y, 59y	CFH c.1520-1G>A p.(Glu566Ala) het.	Deceased donor [58y]	Moderna<br>Moderna	306 [9]<br>226 [8]	266 [9]<br>269 [8]	1.42 [9]<br>1.28 [8]	84 [9] | 71 [9]<br>80 [8] | 91 [-9]	0.48 [12] | 0.18 [-19]<br>0.82 [8] | 0.48 [-38]	3rd vaccination without eculizumab
**22**	F, 29y, 33y	CFH c.1548T>A p.(Asn516Lys) het.<br>Haplotype CFH-H3 homozygous	Living donor [32y]	Moderna<br>Moderna<br>Pfizer	283 [21]<br>314 [28]<br>268 [57]	158 [26]<br>158 [28]<br>172 [57]	0.81 [21]<br>0.88 [28]<br>0.82 [57]	97 [21] | 97 [-7]<br>87 [28] | 97 [-7]<br>89 [57] | 90 [-2]	0.11 [21] | 0.15 [-7]<br>0.11 [63] | 0.11 [-1]<br>0.23 [57] | 0.25 [-2]	4th vaccination without eculizumab
**23**	M, 33y, 38y	C3 C.481C>T p.(Arg161Trp) het.	Native kidneys	Pfizer [2nd]	227 [6]	184 [6]	1.48 [6]	94 [6] | 87 [-2]	0.07 [6] | 0.06 [-2]	1st vaccination without eculizumab
**24**	F, 17y, 36y	CFH c.1198C>A p.(Gln400Lys) het.<br>MCP c.811_816del (p.Asp271_Ser272del) homo.	Native kidneys	Moderna<br>Moderna	174 [1]<br>237 [24]	170 [1]<br>184 [24]	1.34 [1]<br>0.90 [24]	189 [1] | 209 [-18]<br>218 [24] | 207 [24]	0.49 [1] | 0.29 [-18}<br>0.10 [24] | 0.19 [-25]	1st vaccination without eculizumab
**25**	F, 41y, 50y	CFI c.355G>A p.(Gly119Arg) het.<br>Haplotype CFH-H3 homozygous	Native kidneys	Pfizer<br>Pfizer	233 [12]<br>229 [166]	183 [12]<br>159 [166]	Missing<br>2.30 [166]	208 [12] | 214 [-9]<br>225 [166] | 222 [-2]	Missing<br>Missing	3rd vaccination without eculizumab
**28**	M, 45y, 45y	CFI c.1650_1651del p.(Cys550Trpfs*17) het.	Deceased donor [45y]	Pfizer [3rd]	298 [16]	395 [16]	0.80 [37]	180 [16] | 214 [-2]	Missing	Recently after Ntx
**30**	F, 22y, 29y	CFH c.2572T>A p.(Trp858Arg) het	Native kidneys	Moderna<br>Moderna<br>Pfizer	285 [21]<br>302 [20]<br>234 [21]	214 [21]<br>207 [20]<br>217 [21]	1.58 [21]<br>1.91 [20]<br>2.46 [21]	132 [21] | 146 [21]<br>133 [20] | 132 [-8]<br>149 [21] | 123 [-2][6]	0.19 [21] | 0.23 [-1]<br>0.32 [20] | 0.19 [-8]<br>0.35 [21] | 0.89 [-2]	
**31**	F, 31y, 46y	CFH c.2034g>t p.(Trp678Cys) het.	Living donor [41y]	Moderna<br>Moderna<br>Pfizer<br>Pfizer	154 [28]<br>154 [2][5]<br>153 [8]<br>135 [24][7]	291 [28][7]<br>291 [2][5,7]<br>288 [8][7]<br>325 [24][7]	0.15 [28][7]<br>0.15 [2][5,7]<br>0.10 [8][7]<br>0.10 [24][7]	175 [28] | 166 [28]<br>175 [2] | 166 [-26][5]<br>162 [8] | 164 [-20]<br>206 [24] | 181 [-2]	0.10 [28] | 0.13 [0]<br>0.10 [2] | 0.13 [-26][5]<br>0.19 [8] | 0.26 [-20]<br>0.11 [24] | 0.17 [-2]	Chronic Coombs negative hemolysis due to tacrolimus toxicity (CH50<10%)
**32**	M, 7y, 36y	C3 C.481C>T p.(Arg161Trp) het.<br>Haplotype MCP*ggaac* homozygous	Living donor [31y]	Moderna<br>Moderna	144 [29]<br>144 [8][5]	162 [29]<br>162 [8][5]	0.56 [29]<br>0.56 [8][5]	252 [29] | 259 [-6]<br>252 [8] | 259 [-27][5]	0.11 [29] | 0.15 [-6]<br>0.11 [8] | 0.15 [-27][5]	
**33**	M, 39y, 47y	C3 C.481C>T p.(Arg161Trp) het.	Living donor [42y]	Pfizer<br>Pfizer	306 [21]<br>303 [20]	174 [21]<br>175 [20]	2.72 [21]<br>2.98 [20]	222 [21] | 212 [-7]<br>175 [20] | 222 [-8]	0.28 [21] | 0.32 [-7]<br>0.30 [20] | 0.28 [-8]	
**34**	F, 32y, 52y	CFH c.1520-1G>A p.(?) het.	Deceased donor [45y]	Moderna<br>Moderna<br>Pfizer<br>Pfizer	160 [19]<br>146 [40]<br>154 [42]<br>149 [5]	204 [19]<br>202 [40]<br>198 [42]<br>231 [5]	0.51 [19]<br>0.53 [40]<br>0.54 [42]<br>0.75 [5]	162 [19] | 172 [-9]<br>161 [20] | 171 [-9]<br>155 [42] | 150 [-10]<br>159 [5] | 158 [-24]	0.08 [19] | 0.10 [-9]<br>0.09 [20] | 0.08 [-9]<br>0.15 [42] | 0.12 [-10]<br>0.11 [18] | 0.16 [-24]	

	**35**	F, 28y, 36y	CFH-CFHR1 hybrid protein	Deceased donor [30y]	Pfizer<br>Pfizer<br>Unknown	297 [<30]<br>289 [<30]<br>283 [<30]	Missing<br>230 [<30]<br>Missing	Missing<br>Missing<br>Missing	151 [<30] | 139 [>-30]<br>132 [<30] | 139 [>-30]<br>122 [<30] | 138 [>-30]	Missing<br>Missing<br>Missing	Exact dates of vaccinations unknown
	**36**	M, 0y, 20y	CFH-CFHR1 hybrid protein<br>Haplotype MCP*ggaac* homozygous	Living donor [18y]	Moderna<br>Moderna	202 [9]<br>272 [10]	161 [9]<br>175 [10]	0.9 [9]<br>1.50 [10]	170 [9] | 190 [-5]<br>189 [10] | 170 [-18]	Missing<br>Missing	

[1] Age at first episode of aHUS
[2] Age at first vaccination
[3] Number of days after vaccination
[4] Last value before vaccination
[5] Value(s) after this vaccination is(are) of the same date as value(s) after the previous vaccination
[6] ≥20% increase in serum creatinine (sCr) values
[7] ≥2 of the TMA criteria

Supplementary Figure 1. Overview of serum creatinine, proteinuria, and TMA parameters over time in patients with dubious aHUS relapse

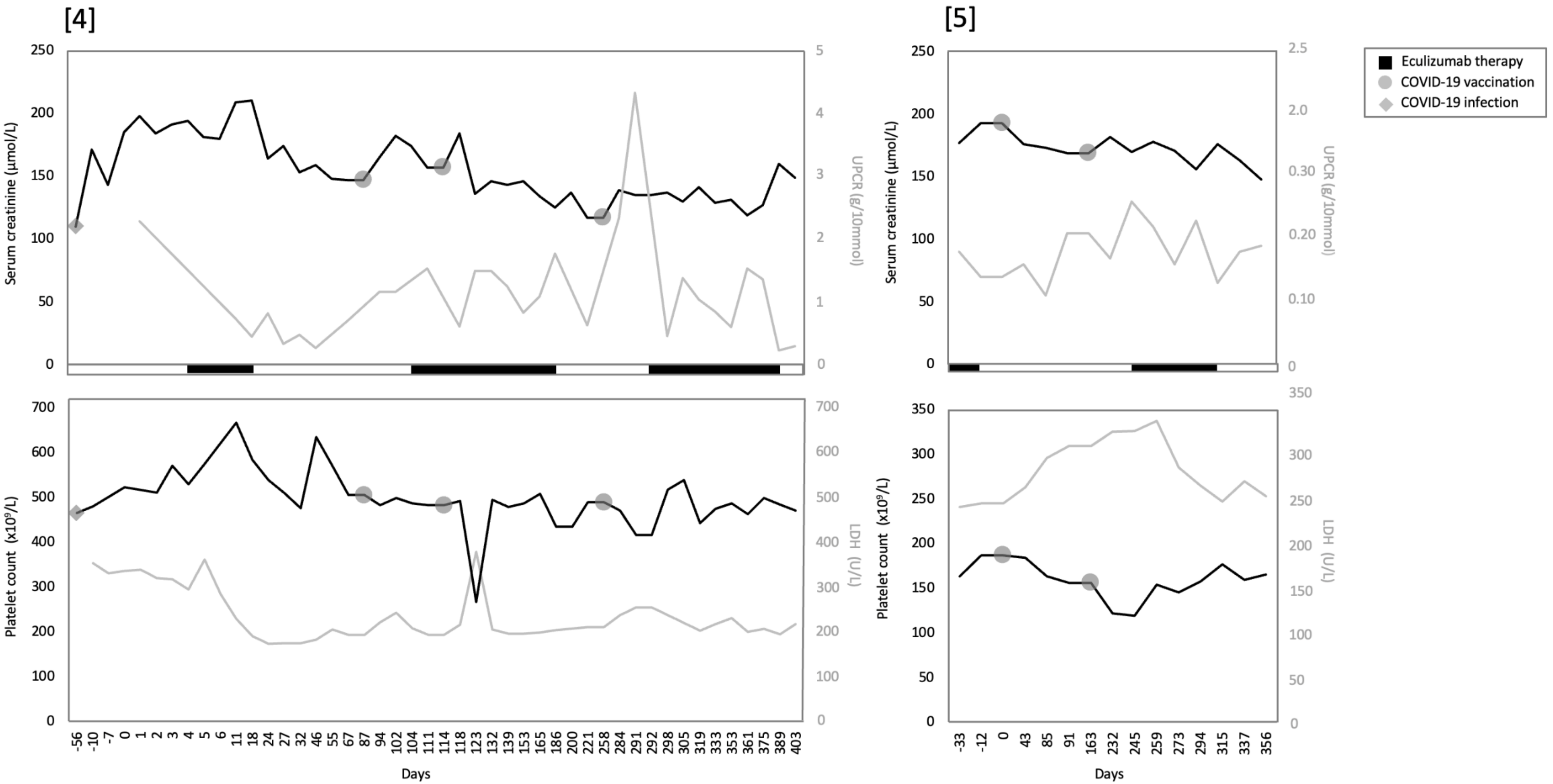

Legend to Supplementary Figure 1

Supplementary Figure 1: Overview trend of serum creatinine, proteinuria and TMA parameters LDH and thrombocytes (platelet count).
